# Efficacy and safety of hydromorphone for postoperative patient-controlled intravenous analgesia for patients undergoing orthopedic surgery: a randomized, double-blinded controlled trial

**DOI:** 10.3389/fmed.2025.1567328

**Published:** 2025-04-28

**Authors:** Qi Wang, Yuanyuan Zhao, Bin Ling, Xiangxiang Chen, Yayun Xie, Haibo Zhao, Jiangang Zhang, Wei Wang, Jie Lv

**Affiliations:** ^1^Department of Anesthesiology, The Affiliated Jiangning Hospital of Nanjing Medical University, Nangjing, China; ^2^Department of Anesthesiology, Huainan First People’s Hospital, The First Affiliated Hospital of Anhui University of Science and Technology, Huainan, China

**Keywords:** hydromorphone, patient-controlled intravenous analgesia, orthopedic surgery, efficacy, safety

## Abstract

**Background:**

The study aimed to evaluate the efficacy and safety of hydromorphone in postoperative patient-controlled intravenous analgesia (PCIA) for orthopedic surgery patients, offering a reference for postoperative pain management in this patient population.

**Methods:**

This was a prospective, randomized, double-blinded, controlled trial involving 80 patients aged 23 to 64 years undergoing elective orthopedic surgery. All participants were randomly assigned to the test group (Group H) and the control group (Group C) by the random number table method. In Group H, hydromorphone (0.2 mg/kg) and palonosetron (4 μg/kg) diluted to 150 mL with saline were used for PCIA, while in Group C, sufentanil (2 μg/kg) and palonosetron (4 μg/kg) were diluted to the same volume. Postoperative pain was assessed using the resting Visual Analog Scale (VAS) at 2, 6, 12, 24, and 48 h postoperatively. The total and effective PCIA button presses within 48 h, along with the number of remedial analgesia cases, were recorded. Ramsay, Awakening time, extubation time, hospital stay duration, and adverse events within 48 h postoperatively were also recorded.

**Results:**

Compared to Group C, Group H had significantly lower VAS scores at 2 and 6 h, as well as Ramsay, SDS, and PSQI scores at 24 and 48 h postoperatively (all *p* < 0.01). Furthermore, the incidence of dizziness and drowsiness within 48 h postoperatively was significantly reduced in Group H (*p* = 0.007 and *p* = 0.003, respectively).

**Conclusion:**

Hydromorphone-based PCIA enhances early postoperative pain relief in orthopedic surgery patients, alleviates postoperative depression and sleep disturbances, and reduces the incidence of dizziness and drowsiness.

**Clinical trial registration:**

This study was registered in the Chinese Clinical Trial. Registry (www.chictr.org.cn) on 01/04/2024 (ChiCTR2400082567).

## 1 Introduction

Postoperative pain is a common complication in orthopedic surgery, significantly affecting patient recovery and quality of life. Patient-controlled intravenous analgesia (PCIA) maintains the minimum effective plasma concentration of analgesics through a “background infusion + patient-controlled” mode, ensuring optimal analgesic effects. This approach is widely used in postoperative pain management (1), labor analgesia (2), and cancer pain management (3). Currently, opioids are the preferred drugs for PCIA due to their superior analgesic effects compared to non-steroidal anti-inflammatory drugs (NSAIDs) and α_2_-receptor agonists. However, opioid use is associated with dose-dependent adverse effects including respiratory depression, nausea, vomiting, dizziness, drowsiness, urinary retention, constipation, pruritus, and potential addiction (4, 5). Thus, an ideal opioid analgesic for PCIA is still lacking, requiring strict dose control.

As a representative of opioids, morphine has been widely used in cancer pain analgesia and postoperative analgesia (6). Hydromorphone, a semi-synthetic morphine derivative, has a ketone group at the 6th position of the benzene ring instead of a hydroxyl group, making it approximately 10 times more potent than morphine and enhancing its distribution in the brain (7). Because of the ketone group at the 6th position, hydromorphone undergoes glucuronidation only at the 3rd position, with the 3-glucuronide exhibiting analgesic and neuroexcitatory effects that are not mediated by opioid receptors. As a result, hydromorphone has stronger analgesic effects and less metabolite accumulation in terms of pharmacodynamics and pharmacokinetics (8, 9). Sufentanil, a fentanyl derivative, produces significant analgesic effects by directly binding to *μ* opioid receptors (MOR) in the nociceptive regions of the spinal cord, medulla, and midbrain. Studies have shown that sufentanil is approximately 10 times more potent than fentanyl, and its therapeutic index (26716) is significantly higher than that of morphine (71) and fentanyl (277) (10, 11). Due to its strong analgesia, prolonged duration of action, and high therapeutic index, sufentanil is currently widely used for postoperative analgesia in various surgical patients (12–14). This study aims to compare the efficacy and safety of hydromorphone and sufentanil in PCIA for orthopedic surgery patients, offering insights for optimizing postoperative pain management strategies.

## 2 Methods

### 2.1 Participants

Eighty patients, aged 23–64 years, with a body mass index (BMI) of 20–28 kg/m^2^ and classified as American Society of Anesthesiologists (ASA) physical status Class I–II, undergoing elective orthopedic surgery between May 1st and November 30th, 2024, in the Department of Anesthesiology at the Affiliated Jiangning Hospital of Nanjing Medical University, were enrolled. Exclusion criteria: (1) mental or neurological diseases, hearing or language disorders; (2) diabetes mellitus, cardiac, pulmonary, hepatic or renal insufficiency; (3) immune or hematological system diseases disorders; (4) history of chronic pain; (5) medical history of psychotropic or analgesic medication; (6) history of alcohol dependence or drug use; (7) history of depression or preoperative self-rating depression scale (SDS) scores ≥ 50; (8) long history of sleep disturbance or preoperative Pittsburgh Sleep Quality Index (PSQI) scores > 5; (9) duration of operation longer than 5 h; (10) postoperative awakening time longer than 2 h; (11) postoperative transfer to intensive care unit (ICU).

### 2.2 Sample size estimation

**Figure 1:**
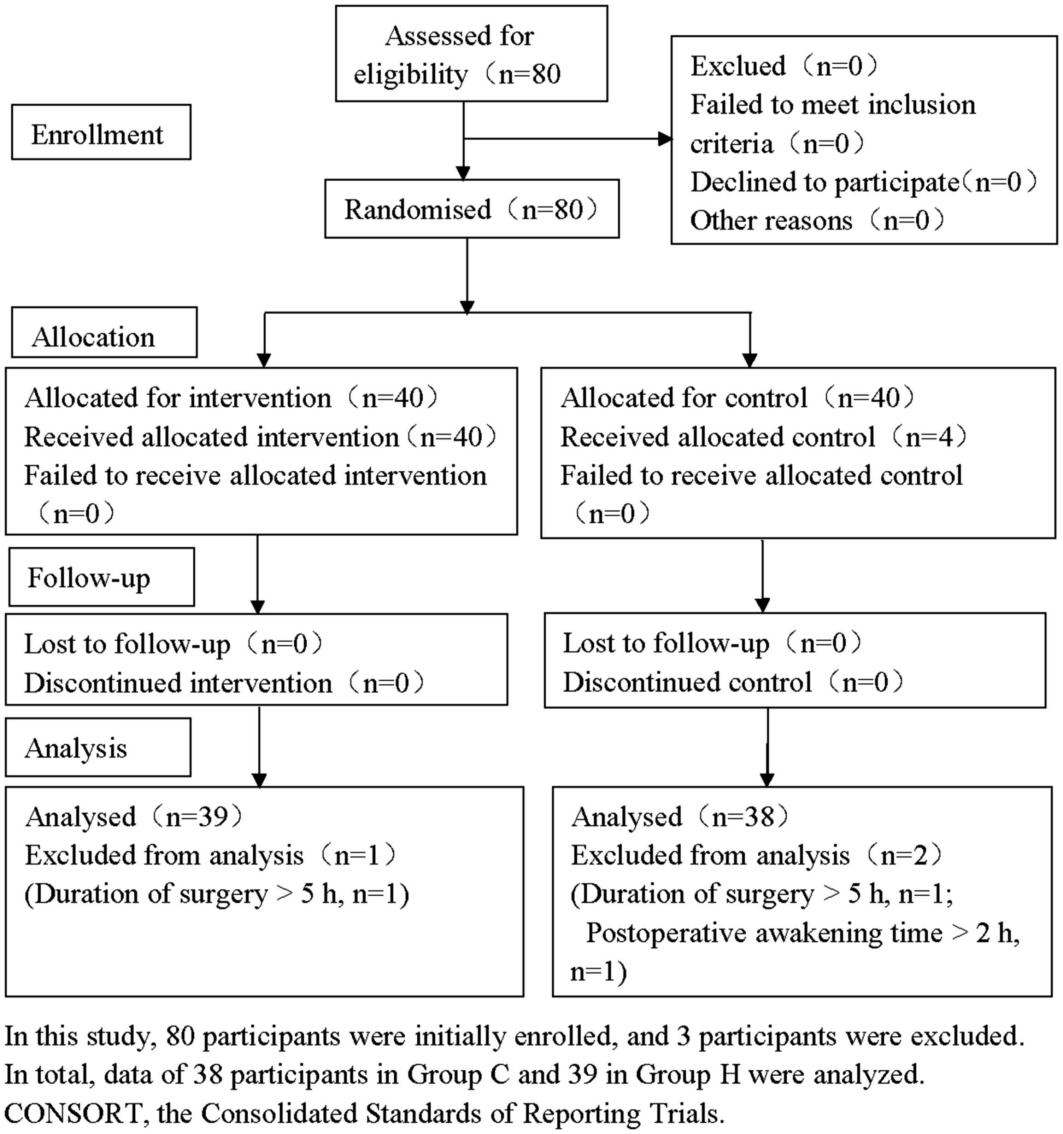
CONSORT flow diagram. In this study, 80 participants were initially enrolled, and 3 participants were excluded. In total, data of 38 participants in Group C and 39 in Group H were analyzed. CONSORT, the Consolidated Standards of Reporting Trials.

Based on previous studiess (15–17) and the results of a preliminary experiment (10 participants in each group), the average VAS scores at 6 h after operation can be reduced about 0.8 (42.1%) in Group H (1.1 ± 0.3 vs. 1.9 ± 0.6, respectively). With *α* = 0.05 and a power value of 80%, a minimum sample size of 36 participants per group was required. Assuming a 10% dropout rate, 40 participants per group were planned for this study. Figure 1 presents the CONSORT flow diagram of the study participant’s recruitment.

### 2.3 Randomization and allocation concealment

Patients were randomly assigned to one of the two groups using random tables generated in SPSS 26.0. A statistician not involved in the study prepared 80 sealed envelopes. Neither observers nor participants were informed of group assignments in this study. To ensure allocation concealment, numbers were stored in sealed, opaque envelopes opened by an independent anesthesiologist not involved in the study. The same orthopedic surgical team conducted all procedures and remained unaware of group assignments. The same anesthesiologist, blinded to group assignments, conducted the postoperative follow-up.

### 2.4 Interventions

Upon completion of surgery, patients in Group C received 0.05 μg/kg sufentanil, and those in Group H received 5 μg/kg hydromorphone, both diluted to 5 mL of normal saline and administered intravenously before connecting PCIA. In Group H, hydromorphone (0.2 mg/kg) and palonosetron (4 μg/kg) diluted to 150 mL with saline were used for PCIA, while in Group C, sufentanil (2 μg/kg) and palonosetron (4 μg/kg) were diluted to the same volume. Both groups received a background infusion rate of 2 mL/h, a bolus dose of 1 mL, a lockout interval of 15 min, and a total duration of 48 h.

### 2.5 Anesthesia management

All patients were instructed to fast for 12 h and refrain from drinking liquids for 6 h before surgery, with no preoperative medication administered. Upon entering the operating room, a peripheral venous access was established, and routine monitoring, including ECG, heart rate (HR), oxygen saturation (SpO₂), non-invasive blood pressure (NIBP), end-tidal carbon dioxide (PETCO₂), and bispectral index (BIS), was conducted. Radial artery catheterization was performed under local anesthesia to monitor invasive arterial pressure (IAP). After 5 min of mask oxygenation (8 L/min), midazolam (0.05 mg/kg), propofol (1.5 mg/kg), cisatracurium (0.2 mg/kg), and sufentanil (0.4 μg/kg) were injected sequentially to induce of general anesthesia. After tracheal intubation, mechanical ventilation was performed with the following settings: IPPV mode; tidal volume (VT) of 8 mL/kg; respiratory rate (RR) of 14 breaths/min; an inspiration-expiration ratio (I:E) of 1:2; and an inhaled oxygen concentration of 70%. Intraoperative P_ET_CO_2_ was maintained between 35 mmHg and 45 mmHg by adjusting mechanical ventilation parameters. Anesthesia was maintained using intraoperative target controlled infusion (TCI) of propofol (plasma target concentration of 1.0–3.0 μg/mL) and remifentanil (plasma target concentration of 1.0–3.0 ng/mL). Cisatracurium was intravenously infused at a dose of 0.1–0.2 mg/kg/h, and the BIS value was maintained between 40 and 60. During surgery, hypertension (MAP increased by 20% from baseline or SBP > 160 mmHg) was treated with 12.5 mg intravenous urapidil; hypotension (MAP decreased by 20% from baseline or SBP < 90 mmHg) with 100 μg intravenous phenylephrine; tachycardia (HR > 100 beats/min) with 30 mg intravenous esmolol; and bradycardia (HR < 50 beats/min) with 0.25 mg intravenous atropine. Cisatracurium infusion was stopped 30 min before surgery, while propofol and remifentanil were discontinued at the final skin suture. Patients were transferred to the postanesthesia care unit (PACU) for recovery. Extubation was performed once patients regained consciousness, muscle strength, swallowing reflex, and spontaneous breathing, with a V_T_ > 5 mL/kg, respiratory rate >12 breaths/min. Patients with a Steward score of >4 points (18) were transferred to the ward. If patients had VAS scores >3 points within 48 h postoperatively and did not experience relief by pressing the PCIA pump, 0.5 mg/kg ketorolac was administered for remedial analgesia, and the number of remedial cases was recorded.

### 2.6 Outcomes

The primary outcomes were resting VAS scores at 6 h postoperatively and the incidence of dizziness and drowsiness within 48 h postoperatively.

The secondary outcomes included: (1) resting VAS scores at 2, 12, 24, and 48 h postoperatively; (2) total and effective PCIA button presses, and the number of remedial analgesia cases within 48 h postoperatively; (3) postoperative awakening time, extubation time, and hospital stay duration; (4) SDS and PSQI scores preoperatively and at 24 and 48 h postoperatively; (5) Ramsay scores at 24 and 48 h postoperatively; (6) intraoperative blood loss, infusion volume, and consumption of propofol and remifentanil; (7) the incidence of postoperative nausea and vomiting, skin pruritus, and respiratory depression (SpO₂ < 90%) within 48 h postoperatively.

The Ramsay sedation scale criteria were as follows: 1 point - anxiety, excitement, or restlessness; 2 points - cooperative, obedient, and quiet; 3 points - asleep but responsive to commands; 4 points - asleep but responsive to mild shaking or loud voice; 5 points - asleep but responsive to painful stimuli (e.g., firm pressure on the nail bed); 6 points - asleep and unresponsive to any stimuli. A score of 1 point indicated inadequate sedation, 2–4 points indicated appropriate sedation, and 5 or 6 points indicated excessive sedation (19).

The SDS scoring system consisted of 20 items, each rated on a four-point scale based on symptom frequency. The total score was multiplied by 1.25 to yield a standardized score. An SDS score of <50 points was considered normal (20).

The PSQI assessment included seven components: subjective sleep quality, sleep latency, sleep duration, sleep efficiency, sleep disturbances, use of hypnotic drugs, and daytime dysfunction. Each component was scored from 0 to 3 points, with a total score ranging from 0 to 21 points. A PSQI score of ≤5 points indicated good sleep quality, while a score of >5 points suggested poor sleep quality and potential sleep disturbances (21).

### 2.7 Statistical analysis

Data analysis was performed using SPSS version 26.0 (SPSS Inc., Chicago, IL, United States). The normal distribution of continuous variables was tested using Shapiro–Wilk test. Data that conform to the normal distribution were presented as mean ± standard deviation (SD), and differences between the two groups were analyzed using an independent samples t-test. The comparison of different points in the group was carried out by Bonferroni test. Categorical variables are presented as frequencies (%), and differences between the two groups were analyzed using the χ^2^ test or Fisher’s exact test. A *p*-value < 0.05 was considered statistically significant.

## 3 Results

### 3.1 Enrollments of participants

In this study, 80 participants were initially screened, and 3 were excluded: in Group C, one participant was excluded due to surgery duration >5 h, and another due to postoperative awakening time >2 h; in Group H, one participant was excluded due to surgery duration >5 h. A total of 38 participants in Group C and 39 in Group H were included in the statistical analysis (Figure 1).

### 3.2 Baseline characteristics

Patients in both groups had similar demographic characteristics, including age, gender, body mass index (BMI), ASA grade, surgical category, surgery duration, intraoperative blood loss, infusion volume, and consumption of propofol and remifentanil (all *p* > 0.05; Table 1).

**Table 1:** Patient demographic and perioperative data.

Variable	Group H (*n* = 39)	Group C (*n* = 38)	*p*-value
Age, year (mean±SD)	45.1 ± 6.7	43.9 ± 7.4	0.526
Gender, n(%)			0.338
Male	22 (56.4)	20 (52.6)	
Female	17 (43.6)	18 (47.4)	
BMI, kg/m^2^ (mean±SD)	23.6 ± 3.3	25.2 ± 4.1	0.279
Class of ASA, n(%)			0.588
I	15 (38.5)	13 (34.2)	
II	24 (61.5)	25 (65.8)	
Surgery category, n(%)			
Open reduction and internal fixation of radial fracture	8 (20.5)	9 (23.7)	0.926
Open reduction and internal fixation of humeral fracture	6 (15.4)	4 (10.5)	0.733
Open reduction and internal fixation of femoral fracture	11 (28.2)	10 (26.3)	0.852
Open reduction and internal fixation of tibial fracture	10 (25.6)	9 (23.7)	0.618
Open reduction and internal fixation of lumbar fracture	4 (10.3)	6 (15.8)	0.816
Duration of surgery, min (mean±SD)	122.8 ± 14.5	126.1 ± 15.9	0.543
Intraoperative blood loss, ml (mean±SD)	316.8 ± 28.2	302.4 ± 26.3	0.298
Intraoperative infusion volume, ml (mean±SD)	1585.6 ± 47.7	1557.4 ± 45.8	0.728
consumption of propofol, mg (mean±SD)	612.6 ± 31.8	623.8 ± 35.3	0.396
consumption of remifentanil, mg (mean±SD)	1.6 ± 0.7	1.5 ± 0.5	0.508

Data are shown as mean ± SD or n (%). A *p*-value < 0.05 was considered to be statistically significant. SD, standard deviation; BMI, body mass index; ASA, American Society of Anesthesiologists.

### 3.3 Postoperative pain

Compared to Group C, resting VAS scores at 2 and 6 h postoperatively were significantly lower in Group H (*p* < 0.01; Table 2). No significant differences were found between the two groups in resting VAS scores at 12, 24, and 48 h postoperatively, total number of button presses, effective presses, or cases of remedial analgesia within 48 h postoperatively (*p* > 0.01; Table 2).

**Table 2:** Postoperative VAS scores.

Variable		Group H (*n* = 39)	Group C (*n* = 38)	*p-*value
Postoperative VAS scores, score (mean±SD)	2 h	1.0 ± 0.4	1.8 ± 0.5	0.005
	6 h	1.2 ± 0.5	2.1 ± 0.7	0.002
	12 h	1.9 ± 0.8	2.3 ± 0.7	0.688
	24 h	2.1 ± 1.0	2.4 ± 0.9	0.581
	48 h	2.3 ± 0.8	2.4 ± 1.1	0.452
Presses of PCIA within 48 h postoperatively, times (mean±SD)	Total presses	3.8 ± 1.2	4.1 ± 0.8	0.706
	Effective presses	2.1 ± 0.9	2.3 ± 1.1	0.547
Remedial analgesia, n(%)		3 (7.7)	5 (13.2)	0.286

Data are shown as mean ± SD or n (%). A *p*-value < 0.05 was considered to be statistically significant between the groups. SD, standard deviation; VAS, Visual Analog Scale; PCIA, patient-controlled intravenous analgesia.

### 3.4 Ramsay, SDS and PSQI scores

Compared to group C, Ramsay, SDS and PSQI scores at 24 and 48 h postoperatively were significantly lower in group H (*p* < 0.01; Table 3).

**Table 3:** Ramsay, SDS and PSQI scores.

Variable		Group H (*n* = 39)	Group C (*n* = 38)	*p*-value
Ramsay scores, score	24 h postoperatively	2.3 ± 0.8	2.9 ± 0.7	0.008
	48 h postoperatively	2.1 ± 0.5	2.8 ± 0.9	0.005
SDS scores, score	preoperatively	43.8 ± 11.2	41.1 ± 13.6	0.696
	24 h postoperatively	48.3 ± 9.9	59.8 ± 10.7	<0.001
	48 h postoperatively	46.5 ± 9.2	56.6 ± 11.4	0.007
PSQI scores, score	preoperatively	3.9 ± 1.1	4.1 ± 0.8	0.758
	24 h postoperatively	7.6 ± 2.8	12.8 ± 3.5	0.003
	48 h postoperatively	6.1 ± 1.9	10.9 ± 2.7	0.005

Data are shown as mean ± SD. A *p*-value < 0.05 was considered to be statistically significant between the groups. SD, standard deviation; SDS, self-rating depression scale; Pittsburgh Sleep Quality Index.

### 3.5 Postoperative recovery and adverse reactions

Compared to Group C, the incidence of dizziness and drowsiness within 48 h postoperatively was significantly lower in Group H (*p* < 0.01; Table 4). No significant differences were found between the two groups in postoperative awakening time, extubation time, hospital stay, incidence of nausea and vomiting, respiratory depression, or pruritus within 48 h postoperatively (*p* > 0.05; Table 4).

**Table 4:** Postoperative recovery and adverse events.

Variable	Group H (*n* = 39)	Group C (*n* = 38)	*p*-value
Awakening time, min (mean±SD)	19.3 ± 5.8	21.2 ± 6.2	0.776
Time to extubation, min (mean±SD)	22.7 ± 6.4	24.1 ± 5.5	0.858
Hospital stay, min (mean±SD)	8.7 ± 1.3	8.5 ± 1.6	0.310
Dizziness, n(%)	2 (5.1)	8 (21.1)	0.007
Drowsiness, n(%)	0	5 (13.2)	0.003
Nausea and vomiting, n(%)	4 (10.3)	3 (7.9)	0.916
Respiratory depression, n(%)	0	0	
Pruritus, n(%)	1 (2.6)	0	0.545

Data are shown as mean ± SD or n (%). A *p*-value < 0.05 was considered to be statistically significant. SD, standard deviation.

## 4 Discussion

Hydromorphone is a novel opioid analgesic that primarily stimulates *μ*-opioid receptors in the central nervous system (CNS). Its distinct chemical structure, differing from morphine, contributes to its superior analgesic effects (7). In addition, hydromorphone’s relatively strong affinity for *κ* and *δ* receptors contributes to its advantages in treating depression and sleep disorders (22). This study indicates that hydromorphone used for PCIA in postoperative orthopedic patients enhances early postoperative analgesia and alleviates postoperative depression and sleep disturbances.

Sufentanil is widely used in postoperative analgesia due to its strong analgesic effect, long duration and high therapeutic index. Studies have shown that the analgesic efficacy of sufentanil is approximately 1,000 times that of morphine and 100 times that of hydromorphone (10, 11, 14). Studies by Min et al. (23) and Dong et al. (13) demonstrated that higher patient satisfaction was achieved with 2 μg/kg or 3 μg/kg of sufentanil for PCIA after hip and spinal surgeries. Therefore, the control group received 2 μg/kg of sufentanil for PCIA, which was compared with 0.2 mg/kg of hydromorphone in this study.

A meta-analysis involving 13 studies and 812 patients (24) indicated that the median protein binding rate of hydromorphone was 11.6%, with the free fraction remaining almost constant, while that of sufentanil was 88.4%, with the free fraction increasing at the end of patient-controlled analgesia (PCA) (19). As a result, hydromorphone demonstrates superior analgesic efficacy in the early postoperative period. Therefore, the primary outcomes of this study was the VAS score at 6 h after surgery. In this study, resting VAS scores at 2 and 6 h postoperatively were significantly lower in the test group compared to the control group, confirming the superior early analgesic effect of hydromorphone. Additionally, hydromorphone exerts its effects within 5 min of intravenous administration and rapidly distributes to various organs, indicating a faster onset of action compared to sufentanil and morphine (25). Furthermore, the results of this study showed that the incidence of dizziness and drowsiness and Ramsay scores in the test group were significantly lower than those in the control group, which may be related to hydromorphone’s low protein binding rate, stable free fraction, and reduced accumulation (24).

Recent studies have shown that hydromorphone may have a biased effect on the G protein-coupled MOR pathway compared to traditional opioids like morphine and methadone, resulting in enhanced analgesic effects (26). Cellular analysis by Manabe et al. (27) demonstrated that hydromorphone’s internalization effect is weaker than that of MOR agonists (e.g., fentanyl), suggesting that hydromorphone may favor G protein-mediated signaling over receptor-dependent downregulation and desensitization. This analgesic mechanism differs from typical opioids, potentially providing a more durable and stable effect in certain chronic pain conditions. However, no significant difference was observed in resting VAS scores at 12, 24, and 48 h postoperatively between the two groups, likely due to the comparable analgesic doses of hydromorphone and sufentanil used in this study (14).

Studies have shown that *μ*, *κ*, and *δ* opioid receptors play crucial roles in mood regulation, with κ and δ opioid receptor agonists demonstrating favorable antidepressant effects (28–30). Although MOR agonists provide strong analgesic and sedative effects, they may cause adverse reactions such as respiratory depression, pruritus, constipation, and euphoria. κ opioid receptor agonists not only provide analgesic effects, but also inhibit addiction, while δ opioid receptor agonists provide strong analgesic activity and anti-anxiety, depression and organ protection effects (31). Hydromorphone’s higher affinity for κ and δ opioid receptors, compared to morphine and sufentanil, may contribute to its enhanced ability to alleviate postoperative anxiety and depression, thereby improving sleep quality (32). In this study, the hydromorphone group exhibited significantly lower SDS and PSQI scores during the PCIA period compared to the control group, confirming its effectiveness in alleviating depression and sleep disturbances.

This study has several limitations. The outcomes in this study were recorded within 48 h after surgery, and longer observation times may increase the reliability. Additionally, the incidence and severity of postoperative depression and sleep disorders were not thoroughly analyzed in this study. Finally, this study was a single-center small sample size study, and further multi-center large sample size studies were needed in the future.

## 5 Conclusion

In conclusion, hydromorphone used for PCIA in orthopedic surgery patients provides effective early postoperative pain relief, alleviates postoperative depression and sleep disturbances, and reduces the incidence of dizziness and drowsiness. These findings support hydromorphone as an effective postoperative analgesic and offer valuable insights for optimizing pain management strategies.

## Data Availability

The raw data supporting the conclusions of this article will be made available by the authors, without undue reservation.
